# Application of opioid-free general anesthesia for gynecological laparoscopic surgery under ERAS protocol: a non-inferiority randomized controlled trial

**DOI:** 10.1186/s12871-023-01994-5

**Published:** 2023-01-27

**Authors:** Liang Chen, Wensheng He, Xue Liu, Fahui Lv, Yuanhai Li

**Affiliations:** 1 Department of Anesthesiology, The First Affiliated Hospital of Anhui Medical University, Hefei, Anhui People’s Republic of China; 2 Department of Anesthesiology, The Second People’s Hospital of Hefei, Hefei Hospital Affiliated to Anhui Medical University, Hefei, Anhui People’s Republic of China; 3 Department of Obstetrics and Gynaecology, The Second People’s Hospital of Hefei, Hefei Hospital Affiliated to Anhui Medical University, Hefei, Anhui People’s Republic of China

**Keywords:** Opioid-free anesthesia, ERAS, Gynecological Laparoscopic surgery, Non-inferiority, Analgesia

## Abstract

**Background:**

Enhanced recovery after surgery (ERAS) is now widely used in various surgical fields including gynecological laparoscopic surgery, but the advantages of opioid-free anesthesia (OFA) in gynecological laparoscopic surgery under ERAS protocol are inexact.

**Aims:**

This study aims to assess the effectiveness and feasibility of OFA technique versus traditional opioid-based anesthesia (OA) technique in gynecological laparoscopic surgery under ERAS.

**Methods:**

Adult female patients aged 18 ~ 65 years old undergoing gynecological laparoscopic surgery were randomly divided into OFA group (Group OFA, *n* = 39) with esketamine and dexmedetomidine or OA group (Group OA, *n* = 38) with sufentanil and remifentanil. All patients adopted ERAS protocol. The primary outcome was the area under the curve (AUC) of Visual Analogue Scale (VAS) scores (AUC_VAS_) postoperatively. Secondary outcomes included intraoperative hemodynamic variables, awakening and orientation recovery times, number of postoperative rescue analgesia required, incidence of postoperative nausea and vomiting (PONV) and Pittsburgh Sleep Quality Index (PSQI) perioperatively.

**Results:**

AUC_VAS_ was (Group OFA, 16.72 ± 2.50) vs (Group OA, 15.99 ± 2.72) (*p* = 0.223). No difference was found in the number of rescue analgesia required (*p* = 0.352). There were no between-group differences in mean arterial pressure (MAP) and heart rate (HR) (*p* = 0.211 and 0.659, respectively) except MAP at time of surgical incision immediately [(Group OFA, 84.38 ± 11.08) vs. (Group OA, 79.00 ± 8.92), *p* = 0.022]. Times of awakening and orientation recovery in group OFA (14.54 ± 4.22 and 20.69 ± 4.92, respectively) were both longer than which in group OA (12.63 ± 3.59 and 18.45 ± 4.08, respectively) (*p* = 0.036 and 0.033, respectively). The incidence of PONV in group OFA (10.1%) was lower than that in group OA (28.9%) significantly (*p* = 0.027). The postoperative PSQI was lower than the preoperative one in group OFA (*p* = 0.013).

**Conclusion:**

In gynecological laparoscopic surgery under ERAS protocol, OFA technique is non-inferior to OA technique in analgesic effect and intraoperative anesthesia stability. Although awakening and orientation recovery times were prolonged compared to OA, OFA had lower incidence of PONV and improved postoperative sleep quality.

**Trial registration:**

ChiCTR2100052761, 05/11/2021.

**Supplementary Information:**

The online version contains supplementary material available at 10.1186/s12871-023-01994-5.

## Introduction

Gynecological laparoscopic surgery has become the main surgical method of gynecological surgery for its advantages of small trauma, light stimulation to patients and short recovery time. Although generally, laparoscopic technique is less painful than the open one, there are still about 20%-40% of patients have moderate or even severe postoperative pain after laparoscopic surgeies [1]. Postoperative pain can not only stimulate sympathetic nerves, trigger a series of stress responses [2], but also affect neuroendocrine function which results in changes of postoperative mental and psychological state and internal environment disorders.

Opioid is the most commonly used intravenous analgesic, but it can lead to opioid-related side effects such as nausea and vomiting, dizziness, respiratory depression, skin itching, constipation. Serious problems caused by the abuse and misuse of opioid have attracted the attention of international scholars [3]. With the introduction of the concept of “Enhanced recovery after surgery (ERAS)”, it calls for better pain management that minimizes the occurrence of adverse reactions and provides effective analgesia.

ERAS, which was first proposed by Danish surgeon in 1997, is an evidence-based perioperative optimized measure that can reduce surgical stress and inflammatory response, promote rapid postoperative recovery, and improve perioperative safety and comfort by surgeons, nurses, anesthetists and other relevant medical staffs [4]. Under this context, OFA technique comes into being. OFA is a multi-modal anesthesia strategy that combines multiple non-opioid drugs with techniques to obtain high-quality anesthesia [5]. In Jimenez’s study [6], ERAS protocol had several advantages in gynecological laparoscopic surgery without OFA technique. In Massoth and Ziemann-Gimmel’s studies opioid-free anesthesia was researched in PONV [7, 8]. However, there are few studies on the analgesic effect of OFA technique under ERAS, especially in the field of gynecological laparoscopic surgery.

Dexmedetomidine is alpha-2 receptors agonist commonly used with its well sedative effect, and esketamine, an N-Methyl-D-Aspartate (NMDA) receptors antagonist, plays excellent analgesic role in clinic anesthesia. They are both non-opioid drugs. In this study, under ERAS protocol in perioperative period, OFA technique, which contained esketamine and dexmedetomidine, was used in gynecological laparoscopic surgery. This is a non-inferiority randomized controlled trial aiming to compare the OFA and OA technique on the effect of analgesia and sedation, postoperative adverse reaction and postoperative sleep quality among gynecological surgical patients under ERAS protocol. Our results may provide clinical evidence for using OFA technique in the future.

## Materials and methods

### Study design

This prospective, randomized single-centre non-inferiority trial was approved by the Institutional Review Board (IRB) of Hefei Hospital affiliated Anhui Medical University (IRB approved registration number: 2021-KeYan-042) and was registered in the Chinese Clinical Trial Registry (ChiCTR2100052761, 05/11/2021). All methods were carried out in accordance with relevant guidelines and regulations. Written informed consent was obtained from all participants.

### Participants

Adult female patients assessed as American Society of Anesthesiologists Grades I to III undergoing elective laparoscopic hysterectomy for benign causes under general anesthesia between November 2021 and May 2022 were enrolled, aged 18 ~ 65 years old. Exclusion criteria included: a. allergy to study drugs; b. language, mental, or comprehension impairment; c. a history of drug dependence and alcohol abuse; d. preoperative major organ dysfunction; e. pregnancy; f. participated other clinical trials.

### Outcomes

According to Petersen’s study [9], AUC of VAS scores is a suitable indication for evaluating of analgesic effect. We decided that the primary outcome of this study was the AUC_VAS_ from the time of leaving PACU to 48 h after surgery. The secondary outcomes included intraoperative MAP and HR, awakening time and orientation recovery time, number of rescue flurbiprofen axate required, incidence of PONV and perioperative PQSI scores.

### Randomization and blinding

All patients were randomly divided into OFA group (Group OFA) or OA group (Group OA). Randomization was conducted using a computer-generated allocation sequence with a 1:1 allocation by an independent investigator. The randomization sequence was placed in sequentially numbered opaque sealed envelopes and revealed by another investigator after the participants’ enrollment, followed by preparing the study drugs wrapped with opaque paper according to the allocated group and delivered them to the operating room. The result of the group allocation was not released until the time of data analysis. Anesthesia was conducted by an experienced anesthesiologist and the outcome assessments were carried out by another one. Participants, anesthesia implementers and outcome assessors were untold of the group allocation.

### ERAS protocol [6]

1. Carbohydrate-rich diet the day before surgery
2. No mechanical bowel preparation
3. 6 h fast for solids and 2 h fast for clear liquid
4. Maintain euvolemia during surgery
5. Preemptive analgesia
6. Active heating during surgery
7. Postoperative nausea and vomiting prophylaxis
8. Restrictive fluid therapy after surgery
9. Resumption of oral intake of liquids and solids within 24 h after surgery
10. Removal of bladder catheter 12-24 h after surgery
11. Active mobilization on the first postoperative day

### Anesthetic management

All patients undergoing elective gynecological laparoscopic surgery were treated according to ERAS protocol during perioperative period and no preoperative drugs were administered before induction. All patients received total intravenous anesthesia (TIVA) without inhalation.

#### Induction of general anesthesia

Routine monitoring devices were set up to monitor noninvasive blood pressure (BP), electrocardiogram (ECG), heart rate (HR), oxygen saturation (SpO_2_), respiratory rate (RR), partial pressure of end-tidal carbon dioxide (PetCO_2_) and bispectral (BIS) index of patients. In group OFA, patients received dexmedetomidine (0.5 μg/kg i.v.) in a 10-min period before induction followed by a continuous infusion with 0.1–0.3 μg/kg/min until the end of surgery, and in group OA, the same amount of normal saline was administered in the same way. Anesthesia was induced with midazolam (0.05 mg/kg i.v.), propofol (2–2.5 mg/kg i.v.), cis-atracurium (1–1.5 mg/kg i.v.) in all patients. Esketamine (0.3–0.5 mg/kg i.v.) or sufentanil (0.2–0.4 μg/kg i.v.) were administered as analgesics of induction in group OFA and group OA, respectively. I-gel laryngeal mask airway was intubated in all patients after induction followed by setting the ventilation mode of anesthesia machine to intermittent positive pressure ventilation (IPPV) with tidal volume 6-8 ml/kg and respiratory frequency 10–15/min. PetCO_2_ was kept at 35-45 mmHg. After induction the skin was sterilized, ultrasound-guided bilateral transversus abdominis plane (TAP) block was performed by linear probe which was placed in transverse position on the midaxillary line in all patients. The external oblique, internal oblique and transversus abdominis were identified successively followed by using a 100 mm 20G disposable needle punctured the skin. When needle-tip was visualized between transversus abdominis and internal oblique, 15 ml of 0.25% ropivacaine was injected bilaterally.

#### Maintenance of general anesthesia

Intravenous infusion of flurbiprofen axate 50 mg for preventive analgesia were administered 10 min before skin incision in both groups. In group OFA, continuous intravenous infusion of dexmedetomidine 0.1–0.3 μg/kg/min, esketamine 0.3 mg/kg/h [10] and propofol 5–7 mg/kg/h were administered for maintenance of general anesthesia, and continuous intravenous infusion of remifentanil 8–10 μg/kg/h and propofol 5–7 mg/kg/h were administered in group OA. Intermittent bolus intravenous infusion of cis-atracurium was administered for intraoperative muscle relaxation. The infusion rate of drugs was adjusted for maintaining the BIS index at 40–60 and the index of MAP and HR within a 20% range of baseline. Hypotension (MAP < 60 mmHg) was treated with ephedrine 10 mg intravenously and bradycardia (heart rate < 45 bpm) was treated with atropine 0.5 ~ 1 mg intravenously. Hypertension (MAP > 120 mmHg) was treated with urapidil 5 ~ 10 mg intravenously. All drugs were stopped at the end of surgery and intravenous infusion of azasetron 10 mg was administered for preventing PONV.

#### After anesthesia

Patients were transferred to PACU after surgery. When patient was conscious and responsive according to command as well as the BIS index was more than 90, I-gel was removed.

#### Postoperative analgesia and acute pain management

All patients received postoperative controlled intravenous analgesia (PCIA). PCIA protocol of group OFA: esketamine 2.5 mg/kg + flurbiprofen axate 2.5 mg/kg + azasetron 30 mg with total amount of 100 ml and constant infusion rate of 2 ml/h, and in group OA: sufentanil 2 µg/kg + flurbiprofen axate 2.5 mg/kg + azasetron 30 mg were administered with the same PCIA parameters as group OFA. A bolus infusion of flurbiprofen axel 50 mg for rescue analgesia if any patients complained of intolerable pain.

### Observation indicators

#### Demographic data

We collected data on demographic characteristics (including age, BMI, duration of surgery, duration of anesthesia and amount of bleeding).

#### Intraoperative data

The MAP and HR prior to induction (T_1_), surgical incision immediately (T_2_), 30 min after surgical incision (T_3_), at the end of surgery (T_4_), and awakening immediately (T_5_).

#### Postoperative data

##### Recovery time assessment

Awakening time (i.e. time from withdrawal of drugs to patient opening eyes and nodding according to command) and orientation recovery time (i.e. time from withdrawal of drugs to patient’s orientation to time, place and person) were recorded.

##### Postoperative pain assessment

All patients were instructed to use a 0 to 10 cm VAS (“0” representing no pain and “10” representing the worst imaginable pain) preoperatively. VAS scores were collected at the time of leaving PACU (T_6_), 12 h after surgery (T_7_), 24 h after surgery(T_8_), 36 h after surgery (T_9_), 48 h after surgery (T_10_), and the area under the curve (AUC) of VAS scores while coughing from T_5_ to T_9_ were calculated by Graphpad Prism 9.0 software. Number of rescue flurbiprofen axel analgesia required was recorded.

##### Postoperative adverse events

The incidence of PONV during postoperative 48 h were recorded. Nausea refers to an uneasy feeling in the stomach while vomiting refers to the forceful expulsion of gastric contents [11]. The severity of nausea was assessed on a 4-point scale (0 = none, 1 = mild, 2 = moderate, and 3 = severe) by subjective feeling of patients. Rescue anti-emetic (metoclopramide 5 mg i.v.) was available on request if the nausea score was ≥ 2. Patients were treated with 10 mg of metoclopramide in case of two or more vomiting episodes.

##### Perioperative sleep quality assessment

PSQI scores were assessed 1 day before the surgery (T_0_) and 1 month after the surgery (T_11_). The Pittsburgh Sleep Quality Index (PSQI) is a validated, easy, self-administered questionnaire that assesses sleep quality which participants are asked to recall within the past month [12]. PSQI scores > 5 reflects poor sleep quality. It comprises 19 items forming seven subscales, including: a. sleep quality, b. sleep latency, c. sleep duration, d. sleep efficiency, e. sleep disturbance, f. sleep medication, g. daily dysfunction. Each component has a score that ranges from 0 to 3 and PSQI global score ranges from 0 to 21. PSQI score > 5 represents poor sleep quality (PSQ) (Appendix 1: Pittsburgh Sleep Quality Index (PSQI)).

### Sample size calculation

In our pilot study, we enrolled 16 patients randomly being allocated in two groups with 8 patients in each group, and they were not enrolled in the final study. The sample size was calculated by 2-Sample Non-Inferiority test in Power and Sample Size software. The mean AUC_VAS_ was 14.3 and 13.5 in group OFA and group OA, respectively, and standard deviation was 3.0. We assumed that the non-inferiority margin was 2. With 80% power and alpha level of 0.05 (two-tailed), based on the pilot study’s results, the minimum sample size was 78 patients. Considered at least 15% sample drop rate, 90 patients were enrolled finally.

### Statistical analysis

All data were analyzed by SPSS software package (version 26.0; IBM Corporation, Armonk, NY). Continuous data were tested for normality using Shapiro–Wilk test. The independent-samples t test was used for analysis of normally distributed measurement data and non-normally distributed data, which were expressed as median (interquartile range) [M(Q)], were analyzed by non-parametric test. Repeated measures analysis of variance was used for inter-group comparison of MAP and HR. The chi-square test was used for comparison between groups for enumeration data. *P* < 0.05 was defined as statistically significant.

## Results

### Demographic data

**Fig. 1:**
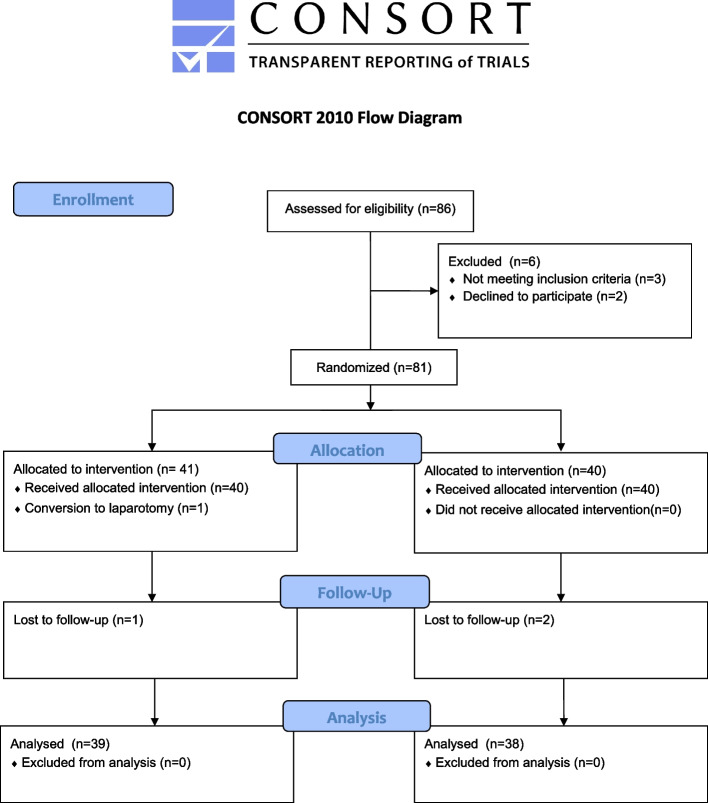
Flowchart based on Consolidated Standards of Reporting Trials (CONSORT) statement

Eighty-six patients were enrolled in this study where three patients met exclusion criteria, two declined to participate, one was converted to laparotomy and three were lost to postoperative follow-up. The remaining 77 patients completed the study (Fig. 1).

There were no significant differences between the two group regarding demographic data included age, BMI, duration of surgery, duration of anesthesia and amount of bleeding (*p* > 0.05) (Table 1).

**Table 1:** Demographic characteristics

**Characteristics**	**Group OFA** **(*n* = 39)**	**Group OA** **(*n* = 38)**	***p* Value**
**Age (yr)**	42.7 ± 12.4	41.5 ± 12.6	0.67
**BMI (kg/cm**^**2**^**)**	22.8 ± 2.9	23.7 ± 2.4	0.13
**During of surgery (min)**	74.4 ± 18.3	81.4 ± 19.3	0.10
**During of anesthesia (min)**	106.6 ± 19.1	112.4 ± 18.8	0.19
**Amount of bleeding (ml)**	73.1 ± 25.8	81.6 ± 27.0	0.16

Data are shown as mean ± SD

*SD* Standard deviation, *BMI* Body mass index

### Primary outcome

**Fig. 2:**
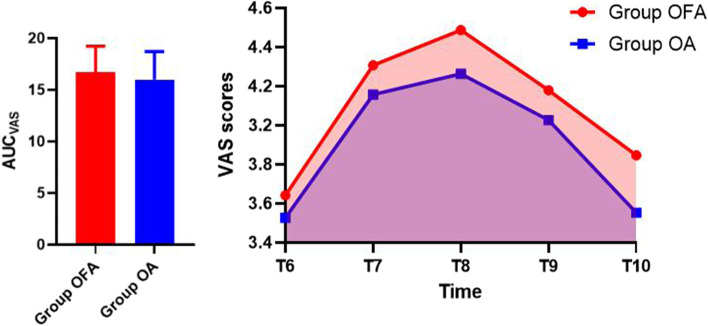
Area under curve(AUC) of VAS scores in two groups (Group OFA, *n* = 39; Group OA, *n* = 38)

AUC_VAS_ were equivalent in the two groups, 16.72 ± 2.50 and 15.99 ± 2.72, respectively (*p* = 0.223) (Fig. 2).

### Secondary outcomes

**Fig. 3:**
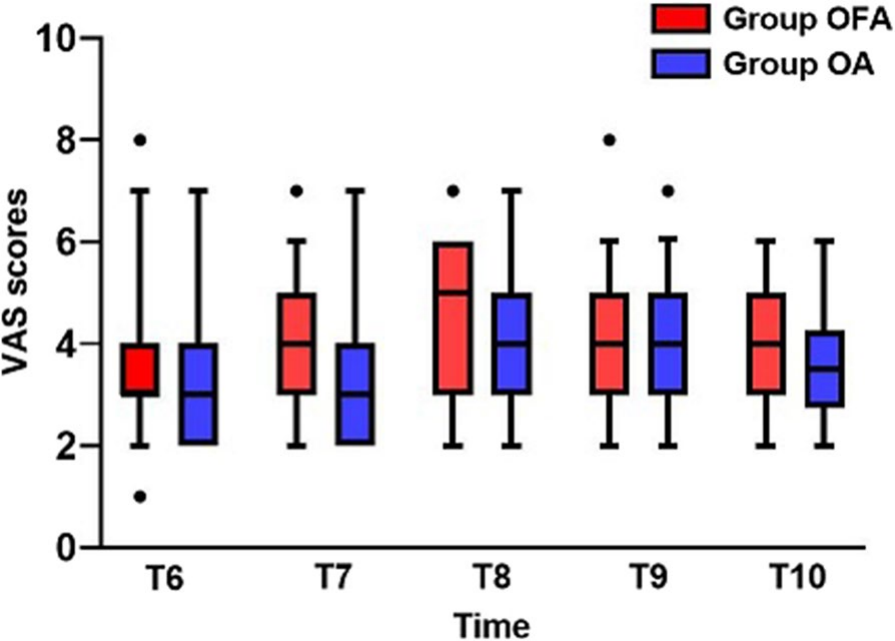
Visual Analogue Scale (VAS) scores from T_6_-T_10_ in two groups (Group OFA, *n* = 39; Group OA, *n* = 38)

We used repeated measures analysis of variance for comparison of VAS from T_6_ to T_10_. Compared to between-subjects effects of VAS, there were no differences in two groups (*p* = 0.193) (Fig. 3). The number of rescue flurbiprofen axate analgesia required in group OFA and group OA were 7 and 4, respectively (*p* = 0.352, RR = 1.859, CI: 0.497–6.960) (Table 2).

**Table 2:** Awakening and orientation recovery time, number of rescue flurbiprofen axate analgesia and incidence of PONV in two groups

**Variable**	**Group OFA** **(*n* = 39)**	**Group OA** **(*n* = 38)**	***p* Value**	**RR** **(95%CI)**
**Awakening time (min)**	14.5 ± 4.2	12.6 ± 3.6	0.04	N/A
**Orientation recovery time (min)**	20.7 ± 4.9	18.5 ± 4.1	0.03	N/A
**Number of rescue analgesia required, n(%)**	7(17.9%)	4(10.5%)	0.35	1.86 (0.49–6.96)
**Incidence of PONV, n(%)**	4(10.3%)	11(28.9%)	0.02	0.283 (0.08–0.89)

Continuous data are shown as mean ± SD, enumeration data are shown as number (percentage)

*SD* Standard deviation, *RR* Risk ratio, *CI* Confidence interval, *N/A* Not applicable

**Fig. 4:**
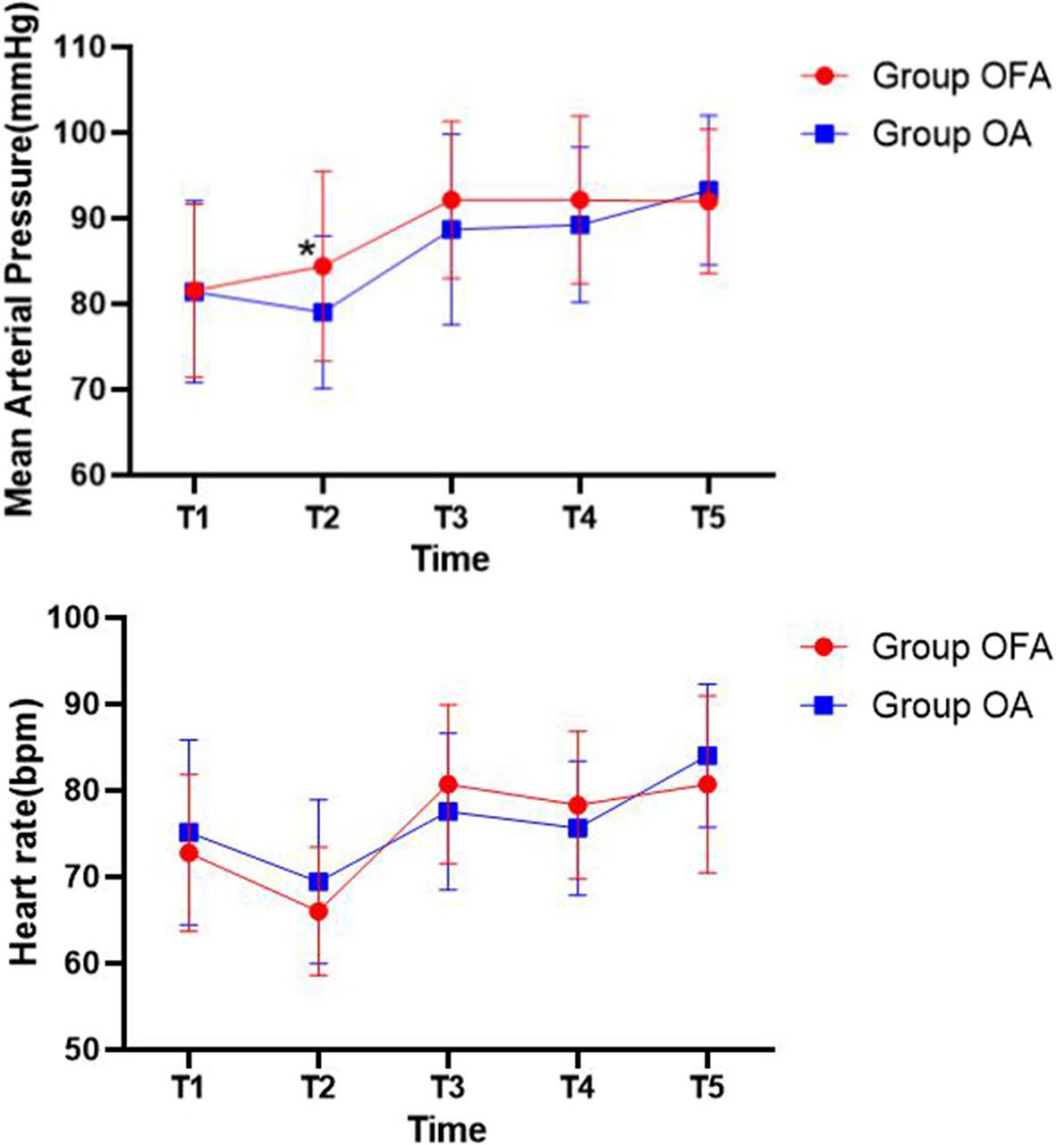
Changes of mean arterial pressure (MAP) and heart rate (HR) from T_1_ to T_5_ in two groups. *p* < .05 is defined statistically significant. *Significant to group OFA (Group OFA, *n* = 39; Group OA, *n* = 38)

We used repeated measures analysis of variance for comparison of MAP and HR from T_1_ to T_5_. MAP in group OFA (84.38 ± 11.08) was higher than that in group OA (79.00 ± 8.92) at the beginning of surgery (*p* = 0.022). As for between-subjects effects of MAP and HR, there were no differences among two groups (*p* = 0.211 and 0.659, respectively) (Fig. 4).

Times of awakening and orientation recovery in group OFA were both longer than which in group OA (*p* < 0.05) (Table 2).

During the first postoperative day, PONV was occurred in 4 patients in group OFA (10.1%) and in group OA, 11 patients reported PONV (28.9%) (*p* = 0.027, RR = 0.283, CI: 0.089–0.896) (Table 2).

Poor Sleep Quality (PSQ) was reported in 6 patients at T_0_ and 3 patients at T_11_ in group OFA, comparing to 5 patients at T_0_ and 2 patients at T_11_ in group OA. We conducted intra-group and between-group comparisons for incidence of postoperative sleep disorder (PSD). Pearson chi-square test was used for comparing incidence of PSQI at T_0_ between two groups (*p* = 0.780, RR = 1.200, CI: 0.333–4.321), continually correction chi-square test was used for other comparisons (Table 3). We used Mann–Whitney U test for comparison of between-group continuous data, and there were no differences in PSQI at T_0_ (*p* = 0.461) and T_11_ (*p* = 0.629). Wilcoxon test was used for intra-group comparison. Postoperative PSQI was improved significantly in group OFA (*p* = 0.007), and in group OA, there was no significant difference (*p* = 0.074) (Table 4).

**Table 3:** Incidence of PSQ in two groups

**Variable**	**Group OFA** **(*n* = 39), n(%)**	**Group OA** **(*n* = 38), n(%)**	***p* Value**	**RR** **(95%CI)**
**T**_**0**_	6(15.4%)	5(13.2%)	0.78	1.20 (0.33–4.32)
**T**_**11**_	3(7.7%)	2(5.3%)	1.00	1.50 (0.23–9.51)
***p*-Value**	0.47	0.42	N/A	
**RR** **(95%CI)**	2.18 (0.50–9.43)	2.72 (0.49–15.02)		

Continuous data are shown as number (percentage)

*RR* Risk ratio, *CI* Confidence interval, *N/A* Not applicable

**Table 4:** PSQI scores in two groups

Variable	**Group OFA** **(*n* = 39), *n*(%)**	**Group OA** **(*n* = 38), n(%)**	***p* Value**
T_0_	4(2)	3.5(2)	0.46
T_11_	3(2)	3(1.25)	0.62
*p* Value	0.01	0.07	N/A

Non-normally distributed data are shown as M(Q)

*M* Median, *Q* Interquartile range, *N/A* Not applicable

## Discussion

In this non-inferiority randomized controlled trial in patients undergoing gynecological laparoscopic surgery, under ERAS protocol, OFA with esketamine and dexmedetomidine was non-inferior to OA with sufentanil and remifentanil in pain scores calculated as the area under the curve for the first 48 h postoperatively. Furthermore, we found that no differences in the intraoperative HR and MAP and the number of rescue analgesia required between two groups, which supports our primary outcome. Meanwhile, lower incidence of PONV and preliminary improvement of PSQ were advantages of OFA which compared to OA. However, in terms of postoperative recovery time, OFA had certain disadvantage.

Although minimally invasive surgery, such as laparoscopic surgery, has become an important part of ERAS in gynecologic surgery, postoperative pain caused by laparoscopic surgery still creates difficulties for anesthesia management in ERAS context. Opioid is gradually replaced by non-opioid medications because of opioid-related disadvantages. In this study, all patients were under ERAS protocol after discussing with surgeon and nurses team. We applied TIVA combined with ultrasound-guided TAP block and non-steroidal anti-inflammatory drug (NSAID) flurbiprofen axate, which was administered for postoperative rescue analgesia. In this context, we purely researched application of opioid and non-opioid medications in gynecological laparoscopic surgery. Patients’ postoperative VAS scores while coughing at different time points were evaluated and recorded. Use of the area under the curve is a common approach to the analysis of continuous variables because of its superiority in precision and bias [13]. In Petersen’s study, they used AUC based on VAS scores while coughing at 0, 2, 4, 6, 8, and 24 h postoperatively in patients undergoing laparoscopic cholecystectomy in day-case surgery to evaluate postoperative pain [9]. In our study, patients’ postoperative hospital days were longer, as well as postoperative pain is a continuous process, so we recorded VAS scores while coughing at 0, 12, 24, 36 and 48 h postoperatively. Compared to VAS at a single time point, AUC_VAS_ can better reflect the overall level of pain for a period of time. Therefore, the result of AUC_VAS_ was effective and intuitional. AUC_VAS_ was no differences in two groups, as well as there was no difference in the number of postoperative rescue analgesia required in two groups, and no differences were found in VAS scores from T_6_-T_10_, therefore we think that analgesic effect of OFA was not inferior to that of OA.

In our study, we administered esketamine and dexmedetomidine for OFA. In a previous placebo-controlled trial [14], esketamine functioned as a longtime analgesic role. An evidence-based review confirmed that as part of ERAS protocol, esketamine improved prognosis of patients [15]. In Massoth’s study, opioid-free protocol as esketamine 0.15 mg/kg for induction and a continuous infusion of dexmedetomidine 0.3 μg/kg/h + esketamine 0.15 mg/kg/h for maintenance of anaesthesia in patients undergoing gynecological laparoscopic surgery was feasible and easy to perform [7].

PONV is another difficulty to be solved in the ERAS context. To reduce PONV, we adopted ERAS protocol which included preoperative carbohydrate loading, limiting fasting of clear liquids, intaking caffeine, chewing gum and postoperative early mobilization [16]. The side-effects of opioids include nausea, vomiting and constipation [17]. In terms of anesthesia, avoidance of intraoperative opioids and volatile anesthetics may be related to reduction of PONV [8]. With the return of patients’ water intake and upcoming PONV pathophysiological climax (24 h postoperatively), PONV occurs more frequently and intensively [18]. In our study, incidence of PONV in group OFA was lower than that of in group OA in a period of 48 h after surgery (10.1%, 28.9%, respectively, *P* = 0.04). Compared to Christina’s study, the incidence of PONV in both groups in our study was lower, the result might preliminarily demonstrate the advantages of ERAS protocol in PONV prophylaxis. However, considered that the simple size of our study was small, this result need to be interpreted with caution. Moreover previous research has proved that dexmedetomidine may reduce the occurrence of PONV while producing sedation and analgesia [19]. Therefore, we can assume that under ERAS protocol, OFA technique in this study has positive effects in reduction of PONV.

PSD, which is related to type of surgery, duration of surgery, methods of anesthesia, anesthesia agents and other factors, has gradually drawn attention. Postoperative pain and opioids have significant effects on postoperative sleep quality. Opioids can lead to postoperative sleep disturbance, which may be related to the regulation mechanism of endogenous opioid activity [20]. Propofol, the interoperative sedative, can reduce the long-term postoperative sleep quality, it may be correlated with the occurrence of postoperative sleep disorders [21]. Esketamine and dexmedetomidine have been proven to improve of sleep quality [22, 23]. PSQI is widely used to assess patients’ sleep condition and provide reference for perioperative management. On the premise of no difference in PSQI before surgery between two groups in this study, we found that 1 month after surgery, PSQI of two groups had no significant difference. But in group OFA, compared to preoperative period, postoperative sleep quality of patients was improved, so we considered that OFA technique may play a role in this. The results of our study can only show that OFA has certain improvement effect on PSD in the short-term period after surgery, but the long-term effect on PSD needs further research.

Times of awakening and orientation recovery in group OFA were longer than those in group OA in this study. Although several studies found that delayed recovery may lead to serious postoperative complications, in this study delayed recovery does not have apparent anesthesia related adverse reactions, the reason may be that our population was ranged from 18–65 years old, which reduced the possibility that elder patients are more susceptible to postoperative complications due to delayed recovery. Furthermore, dexmedetomidine has certain effect on delayed recovery, but its role is gentle and the efficacy is close to the physiological effect of sleep, It is also safe for elder patients [24]. Meanwhile, esketamine was considered does not prolong awakening time in minimally invasive surgery, but the dose-dependent of this drug need to be noticed [25].

Esketamine has the effect of sympathetic activation that can increase heart rate, blood pressure and cardiac output [26], whereas dexmedetomidine plays the opposite role. Add into account the as well as subtle differences of analgesic effect between OFA and OA technique, these factors may lead to our study results regarding MAP and HR. Although MAP in group OFA (84.38 ± 11.08) was higher than that in group OA (79.00 ± 8.92) at T_2_ (*P* = 0.022), no differences were found in total trends of intraoperative hemodynamic variables in both groups. We consider that OFA technique can maintain period of intraoperative anesthesia as smoothly as OA.

There are several limitations in the study. Firstly, although there was significant difference in incidence of PONV between two groups, we didn’t collect the history of PONV and motion sickness, which are also risk factors of PONV. In addition, the number of rescue antiemetics after surgery was not recorded, hence in subsequent study, we will focus on PONV and prepare to observe and record more comprehensive data, even consider testing PONV related blood indicators such as histamine and 5-hydroxytryptamine or serotonin if necessary [27]. Moreover, we think that the sample size was not enough, and the range of age was narrow. Nowadays, more elder patients receive surgery and anesthesia, and the subsequent trauma and stress will bring more serious complications and seriously affect postoperative recovery. In order to carry out OFA technique more comprehensively, we should enroll elder patients, and conduct larger sample and multi-center trial. Thirdly, we should research VAS scores at rest and during movement of patients separately. VAS is a subjective indicator, which would change in accordance to patients’ feeling and understanding of pain. Hence, perhaps combining with serological test, which is related to postoperative pain can be more objective and reliable. In addition, we should prolong the duration of postoperative follow-up, an one-month PSQI follow-up would not completely reflect postoperative sleep quality, and risk factors of PSD should be preoperatively certain. Change of sleep quality is gradual, and a longtime follow-up is necessary.

## Conclusion

Compared to OA technique, OFA technique, which is represented by non-opioid drugs esketamine and dexmedetomidine, may be an alternative anesthesia in gynecological laparoscopic surgery under ERAS protocol with its non-inferiority of analgesic effect, intraoperative anesthesia stability and lower incidence of PONV. Although OFA technique prolonged awakening and orientation recovery time, there was little adverse effect on the quality of anesthesia and recovery. Furthermore, it could improve postoperative sleep quality as a preliminary finding. For better clinical anesthesia effect, ERAS protocol and OFA technique need to be further optimized.

## Supplementary Information

**Additional file 1: Appendix 1.** Pittsburgh Sleep Quality Index (PSQI).

## Data Availability

The datasets used and analysed during the current study available from the corresponding author on reasonable request.
