# Supplementary figures and images for: Application of opioid-free general anesthesia for gynecological laparoscopic surgery under ERAS protocol: a non-inferiority randomized controlled trial
Source: BMC Anesthesiol. 2023 Jan 27;23:34. doi: 10.1186/s12871-023-01994-5 (PMC9881250; doi:10.1186/s12871-023-01994-5)

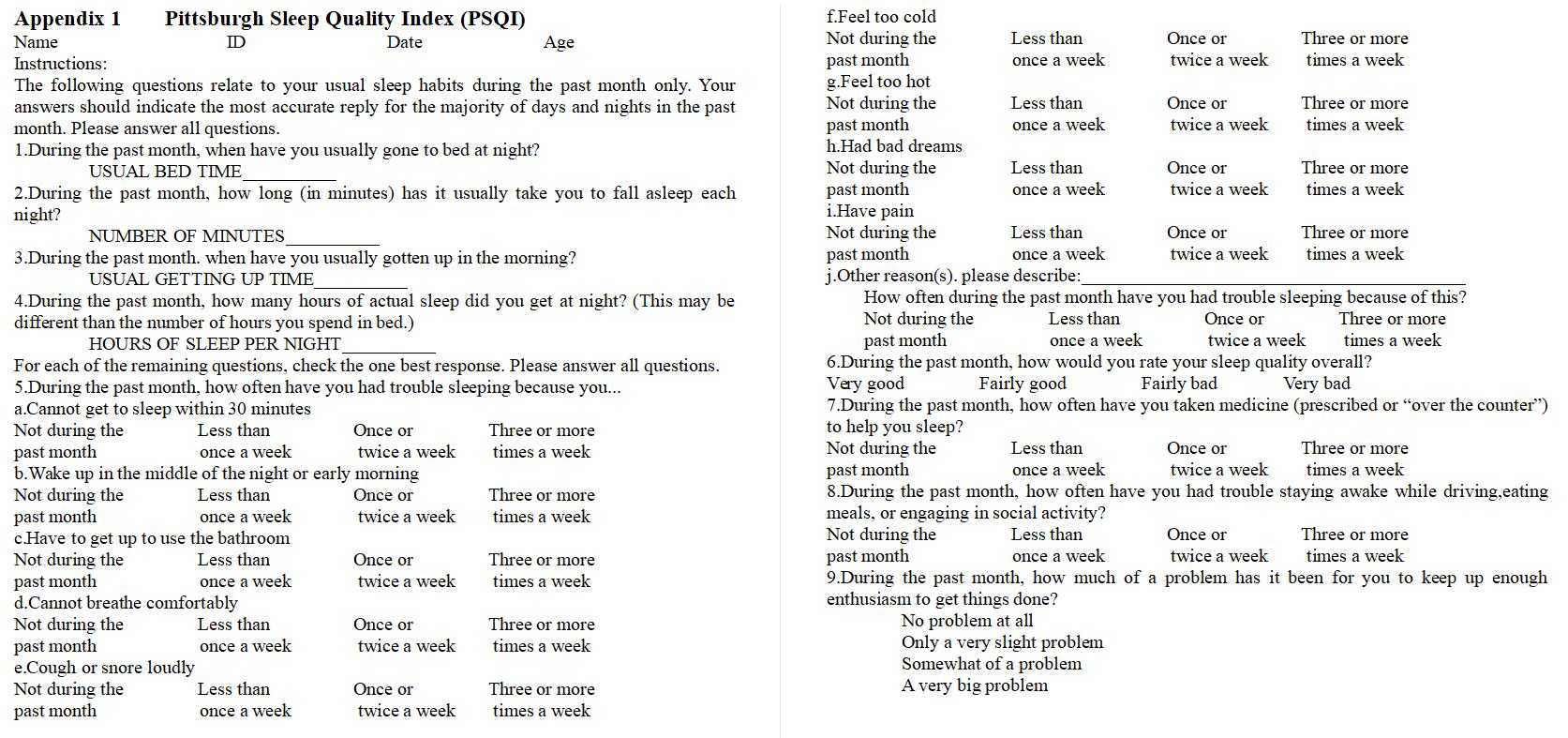


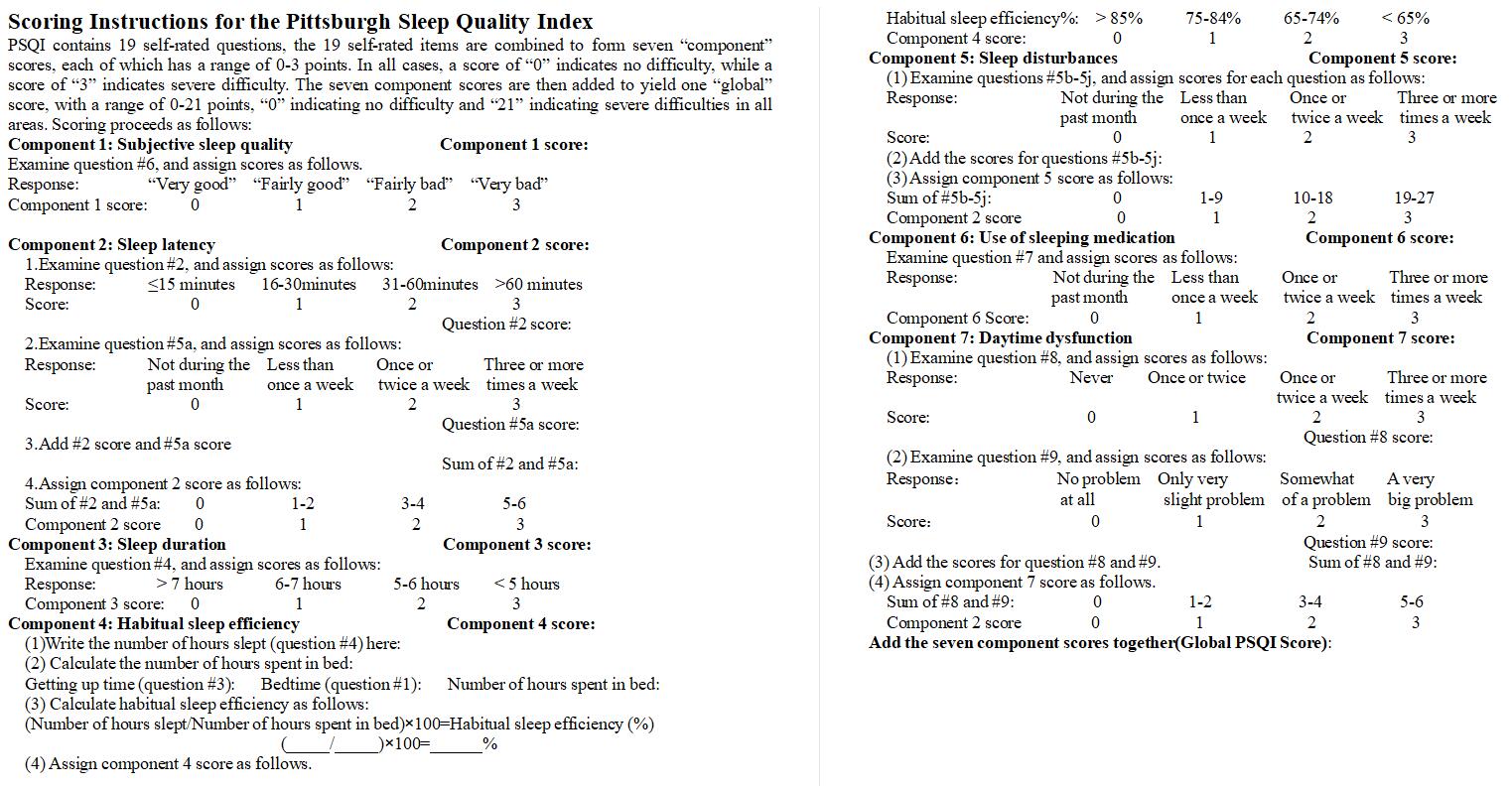

Supplement: Supplementary file 1 — Additional file 1: Appendix 1. Pittsburgh Sleep Quality Index (PSQI). [file 12871_2023_1994_MOESM1_ESM.docx]
